# Optoelectric coordinated modulation of resistive switching behavior in perovskite based synaptic device

**DOI:** 10.1038/s41598-025-88716-8

**Published:** 2025-02-07

**Authors:** Yucheng Wang, Ruixi Huang, Wenyi Zhang, Dingyun Guo, Jiawei Zheng, Hexin Wang, Fobao Huang, Zhuoya Wang, He Guan

**Affiliations:** 1 Research&Development Institute of Northwestern Polytechnical University in Shenzhen, Xi’an, China; 2 School of Microelectronics, Northwestern Polytechnical University, Xi’an, 710072 China

**Keywords:** Perovskite, Memristor, Optoelectric coordinated modulation, Vision recognition, Electrical and electronic engineering, Information storage, Sensors and biosensors

## Abstract

Triple cation halide perovskite (TCP) stands out as a superior photoelectric material, with a broader absorption range, higher absorption efficiency, and improved environmental stability. Due to its excellent synaptic plasticity, TCP facilitates advanced neural morphological operations like light-assisted learning. Here, a modifying layer of polythiophene (P3HT) was incorporated onto the TCP thin film to enhance the resistive switching (RS) characteristics of the synaptic device, which exhibits excellent stability (10^3^ endurance cycles and > 10^3^ s retention time) and low energy consumption (~ 6.3 pJ for electrical stimulus and ~ 6 pJ for optical stimulus). Additionally, the synaptic properties of the perovskite / P3HT heterojunction synaptic device were explored under optoelectric coordinated modulation, encompassing Long-Term Potentiation (LTP), Long-Term Depression (LTD), frequency-dependent plasticity (SRDP) and voltage-dependent plasticity (SVDP). By leveraging the linear characteristics of synaptic plasticity, arithmetic operations, Pavlovian conditioned reflex and vision recognition are achieved. The recognition accuracies of 89.8% / 88.1% (electric synapse) are enhanced to 92.4% / 92.2% after the introduction of optoelectronic cooperative stimulation on the 8 × 8 and 28 × 28 modified national institute of standards and technology (MNIST) handwritten digit datasets. This study holds significant implications for guiding the optoelectronic co-regulation of perovskite synaptic devices in the field of synaptic electronics.

**Supplementary Information:**

The online version contains supplementary material available at 10.1038/s41598-025-88716-8.

## Introduction

The ongoing progression of Moore’s Law has resulted in the von Neumann bottleneck continuing to limit the efficiency of modern computing systems^1^. The advent of neuromorphic computing architectures has been inspired by the functionality of neurons and synapses in the human brain. These architectures introduce in-memory computing capabilities that offer high parallelism, extensive integration, low power consumption, and ultra-fast responsiveness^2^. Systems based on this architectural approach have demonstrated efficacy in enhancing computational efficiency and mitigating the constraints imposed by the von Neumann bottleneck. Among the various components employed in these systems, synaptic devices have attracted considerable attention^3–5^. These neuromorphic devices emulate synaptic behaviour and offer a number of key advantages, including simple structures, diverse resistive switching properties and compatibility with CMOS processes. Consequently, synaptic devices have become a significant area of investigation within the scientific community. To date, a wide range of materials, including oxides^6,7^, chalcogenides^8,9^, organic materials^4,10^, and organic-inorganic hybrid perovskites^5,11–15^, have been investigated for their potential in synaptic device applications. Among these, organic-inorganic hybrid perovskites are particularly noteworthy due to a number of distinctive advantages. Firstly, they combine the cost-effectiveness and ease of fabrication seen in organic materials with the stable performance of inorganic materials^16,17^. Secondly, these perovskites contain mobile ions with low activation energy, making them well-suited for ion migration-based memristive mechanisms^18^. Additionally, their strong light absorption properties introduce a photo-driven effect, further enhancing the functionality of synaptic devices^19^.

Since the introduction of organic-inorganic hybrid perovskites for synaptic device applications in 2015, perovskite synaptic devices have demonstrated considerable potential in the field of optoelectronic applications^20,21^. However, the stability of perovskites as a material presents a significant challenge, as they tend to degrade in high-temperature and high-humidity environments, which limits the further application of these devices^22^. A variety of strategies have been proposed to enhance the stability of these materials, including system-level encapsulation^23^, elemental modification^24^, and interface control^25^. Of these strategies, system-level encapsulation has demonstrated the most substantial impact; however, its intricate processes and elevated costs have impeded its broader implementation. In the context of elemental modification, the utilisation of multi-cationic perovskite structures, comprising combinations of Cs, FA, and MA, has demonstrated efficacy in enhancing material hydrophobicity^26^. Moreover, in the domain of interface control, the utilisation of inorganic materials, such as ZnO, or organic materials, like P3HT, has proven effective in passivating the surface of perovskites^27–29^. For instance, P3HT is an effective method of preventing perovskites from exposure to moisture and oxygen in the air, thus averting degradation in high-humidity and high-oxygen environments. Furthermore, as an organic and transparent passivation layer, P3HT not only aligns with perovskite fabrication processes but also avoids obstructing photo-induced effects^30^. Hence, it is evident that optoelectronic heterojunction synaptic devices based on triple cationic (CsFAMA) halide perovskites /P3HT hold the potential to significantly enhance material stability and boost synaptic device performance.

Presently, synaptic devices rely primarily on a solitary electrical excitation for their operation^31^. However, the introduction of a perovskite synaptic device offers the potential for additional optical excitation. This expansion of possibilities holds significant promise, particularly in terms of reducing power consumption and enabling applications such as vision recognition^32^. Prior to this, it is essential to undertake discrete investigations into the influence of cooperative optoelectronic control in conjunction with electrical control on the RS mechanism and synaptic plasticity of perovskite-based photonic synaptic devices. To address this issue, the present study initiates the fabrication of CsFAMAPbI_xBr_(3-x)/P3HT heterojunction photonic synaptic devices. An analysis was conducted of the perovskite thin films and the characteristics of the synaptic devices. Moreover, the synaptic plasticity and handwritten digit image recognition applications were compared and validated under the influence of single electrical excitation, single optical excitation, and optoelectronic cooperative excitation. This research offers a theoretical insight into the mechanisms and potential applications of perovskite synaptic devices in image recognition under optoelectronic cooperative control.

## Results and discussion

**Fig. 1:**
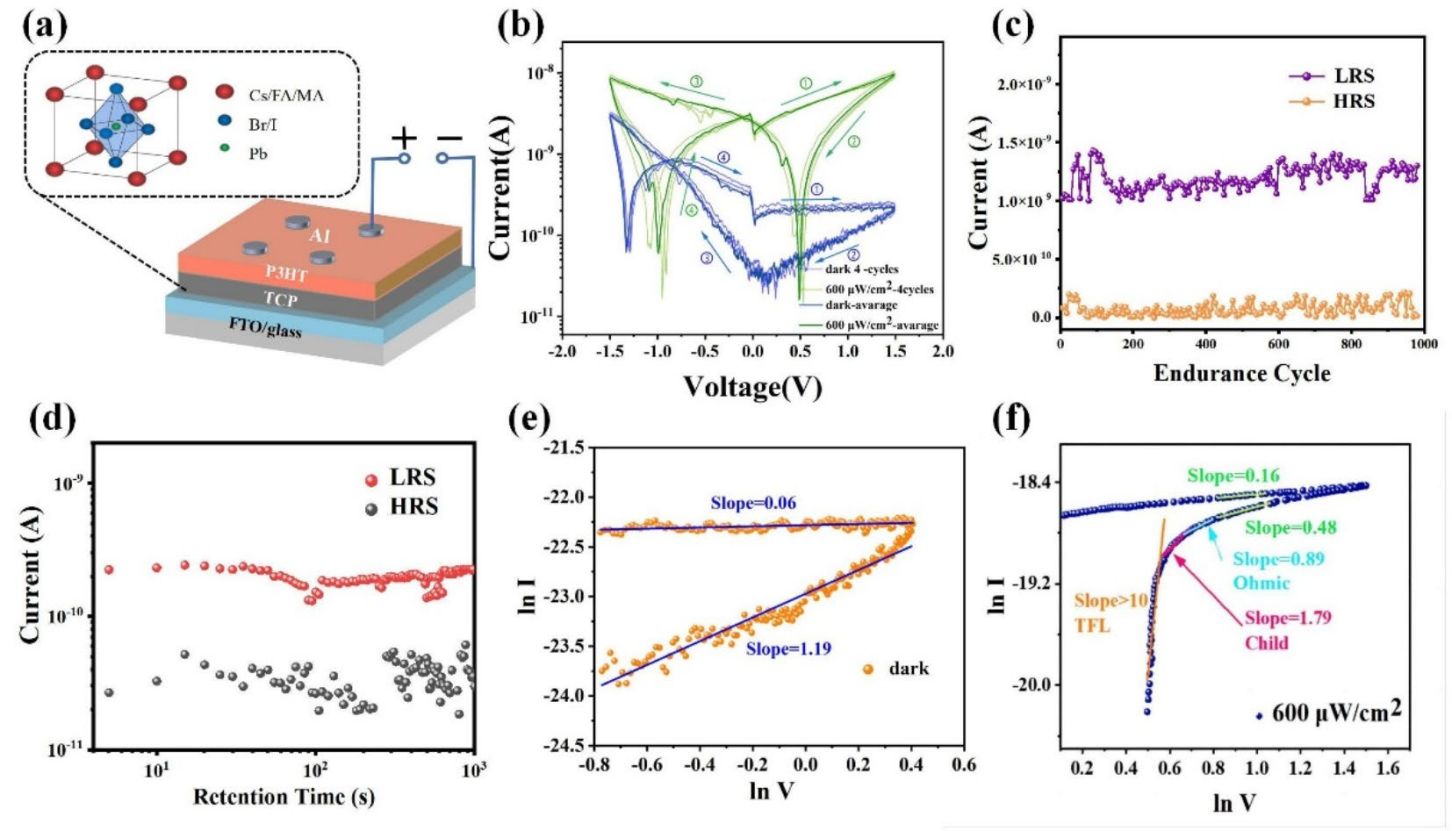
(**a**) Schematic diagram of the Al/P3HT/TCP/FTO synaptic device; (**b**) The I-V characteristic curves of Al/P3HT/TCP/FTO memristors under illumination (600 µW/cm^2^) and dark conditions. The sequence of input voltages is: 0 V → 1.5 V → -1.5 V → 0 V; (**c**) Endurance test of synaptic device for 1000 cycles; (**d**) Retention time of Al/P3HT/TCP/FTO synaptic device; (**e**) Perform piecewise linear fitting on the dark-state I-V curves of the Al/P3HT/TCP/FTO synaptic device, and obtain the slope of each fitted line segment; (**f**) Perform piecewise linear fitting on the dark-state I-V curves of the Al/P3HT/TCP/FTO synaptic device under light conditions, and obtain the slope of each fitted line segment.

The following illustrations present a selection of material characterisation results in conjunction with device schematics. Figure 1**a** depicts the schematic diagram of the Al/P3HT/TCP/FTO synaptic device. As illustrated in **Figure** **S1****c**, the cross-sectional scanning electron microscopy (SEM) image of the TCP/P3HT sample exhibited distinct boundaries, as indicated by the dashed lines. **Figure ****S1****a** illustrates that the root mean square (RMS) value for the TCP/P3HT sample is 127 nm, which is lower than the RMS value of 148 nm for the TCP sample (**Figure** **S1****b**). The majority of defects in the perovskite material are located on the surface and at grain boundaries^33^. This distribution can be attributed to the material’s hydrophilic nature and susceptibility to oxidation. The modification of the TCP film with a P3HT layer serves to partially fill these surface defects and effectively mitigates some of the surface defects and irregularities, resulting in a reduction in surface roughness to a certain extent. **Figure** **S1****d** illustrates the X-ray photoelectron spectroscopy (XPS) spectra of the TCP/P3HT film, which are consistent with the reported literature^34^. **Figure** **S1****e** presents the X-ray diffraction (XRD) spectra for both TCP and TCP/P3HT hybrid layers, confirming the successful synthesis of a highly crystalline CsFAMAPbIBr phase^35^. Furthermore, it is evident that the introduction of the P3HT layer does not alter the crystal structure of the material.

Figure 1b illustrates the current-voltage (I-V) characteristics of the Al/P3HT/TCP/FTO synaptic device under both light (600 µW/cm^2^) and dark conditions. As the voltage input sequence follows 0 V → 1.5 V → -1.5 V → 0 V, the magnitude of the responsive current falls within the range of 10^− 11^ ~ 10^− 9^ (dark state). This value is notably smaller when compared to perovskite resistors lacking the additional modifying layer^32^, which demonstrates the effective reduction of current and power consumption through the incorporation of the P3HT modifying layer. It was observed that the device does not exhibit a sudden change between high and low resistance states, showing excellent analog characteristics. In previous studies, it was demonstrated that the primary resistive switching mechanisms in perovskite synaptic devices originate from the combined effects of interface potential barrier modulation and volume switching halide vacancy conduction filaments^36^. Oxygen vacancies are uniformly distributed within the device, and under the electric field, multiple conduction filaments with consistent directions but varying positions and sizes are formed. These oxygen vacancy conduction filaments gradually break or form in response to changes in the electric field, collectively influencing the device’s resistance. Since these conduction filaments do not break or form simultaneously, the device’s resistance does not experience a sudden change, resulting in a continuous I-V curve. Furthermore, the maximum current contrast between light and dark conditions in the positive voltage region (approximately 10^2^) is significantly larger than that in the negative voltage region (less than 10), indicating that a Schottky barrier is formed due to the difference in Fermi levels between the perovskite and FTO, which hinders iodine vacancy migration^36^. In the positive voltage region, the Schottky heterojunction is reverse biased, thereby limiting the current to a lower level. In the presence of light, photogenerated carriers are produced in the perovskite. At this juncture, in addition to the current originating from the original iodine vacancy migration in the synaptic device, there is also a current generated by the movement of photogenerated carriers. Consequently, both the high-resistance state (HRS) and low-resistance state (LRS) currents are enhanced due to the passage of photocurrent through the junction, while some photogenerated carriers undergo recombination with defects. Furthermore, it can be observed that the I-V curves under illumination are nearly symmetric for positive and negative voltages, whereas in the dark, this symmetry is absent. This is likely due to the reduction of the Schottky barrier between TCP and FTO under illumination, thereby suppressing the device’s rectification characteristics.

**Fig. 2:**
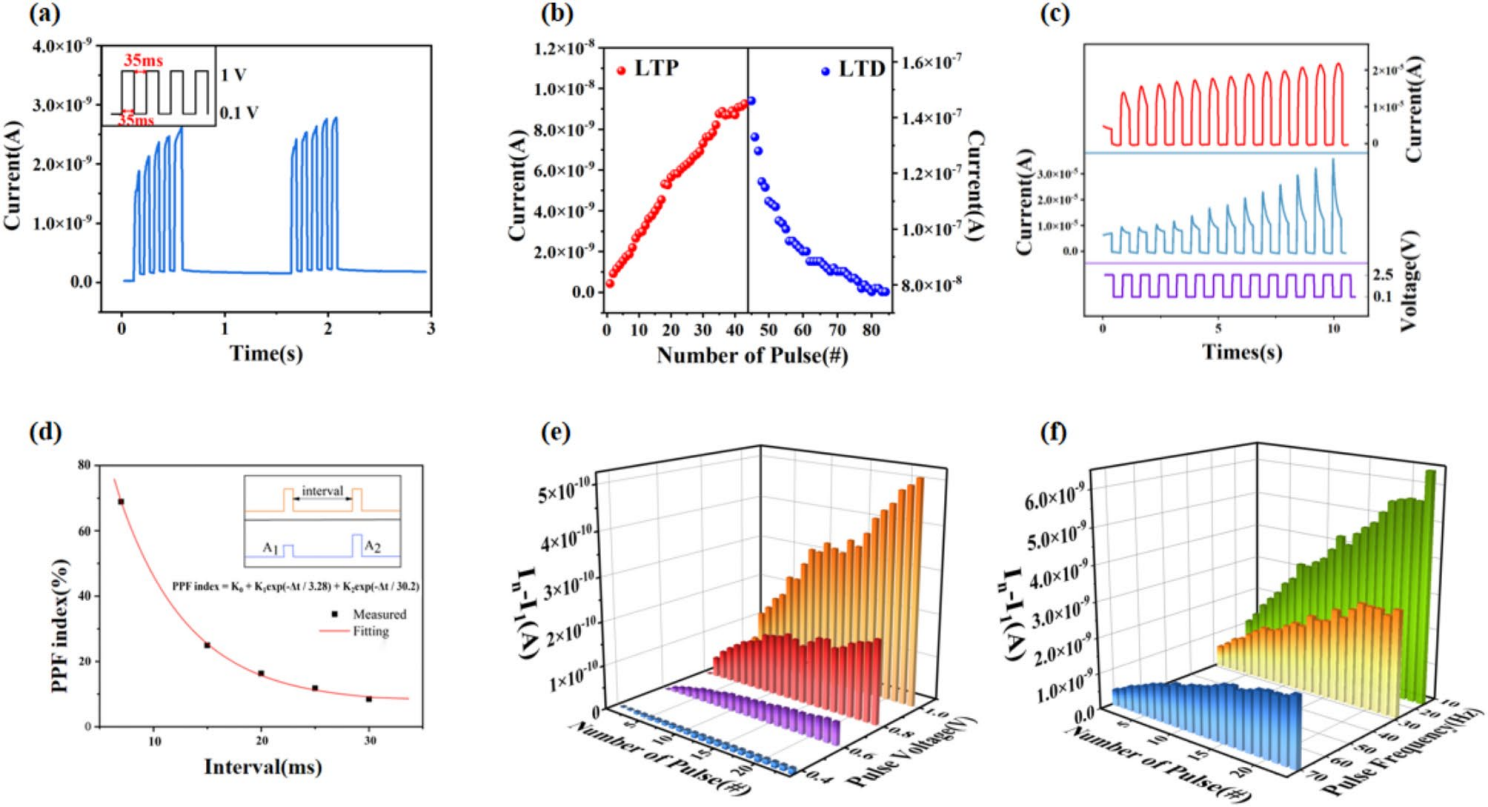
(**a**) EPSC of the Al/P3HT/TCP/FTO synaptic device after 5 consecutive 1 V electrical pulses with a read voltage of 0.1 V; (**b**) The electrical LTP and LTD characteristics of the device; (**c**) EPSC curves under 2.5 V / 0.1 V voltage pulses: the blue I-t curves are under initial scanning, and the red ones are conducted after 100 pulses; (**d**) PPF characteristics in synaptic neurons with dual electrical pulses; (**e**) Comparison of current change magnitude under different pulse voltage amplitudes after I_n_-I_1_ processing when the number of pulses is 23, maintaining the same pulse frequency (28 Hz) and a read voltage of 0.1 V; (**f**)Comparison of current changes under different pulse frequencies after In-I1 processing when the number of pulses is 23, the pulse amplitude is 1 V, and the read voltage is 0.1 V.

In the retention test, the write and read voltages were 1.5 V and 0.1 V, respectively. During the endurance tests, the write voltage and read voltage were 1 V and 0.2 V, respectively, with a pulse width of 15 ms. Figure 1***c***,*** d*** illustrate that the synaptic device demonstrates excellent stability, with an endurance cycle count of 10^3^ and a retention time exceeding 10^3^ seconds. The current has been treated in absolute terms (the same approach is applied in the following sections). Table 1 presents a comparison of the performance of synaptic devices comprising different materials. It can be seen that the structure proposed in this paper performs well in terms of endurance, retention time and energy consumption compared to other structured devices in the table. Figure 1**e**,** f** illustrate the segmented linear fitting of the I-V curve of the synaptic device, thereby obtaining the slope of each fitted segment. As the slope of the lnI-lnV curve under the space-charge-limited conduction (SCLC) mechanism should be greater than or equal to 1^37^, it can be observed from Fig. 2e that the synaptic device does not meet the SCLC mechanism in the dark state. However, under illumination conditions (Fig. 2**f**), as the scanning voltage decreases, the synaptic device meets the Ohmic mechanism, the SCLC mechanism, and the trap-filled limit mechanism (TFL) in sequence, indicating the presence of defect recombination in the synaptic device at this time.

**Table 1:** Comparison of performance of synaptic devices with different materials.

Device Structure	Endurance (No. of cycles)	Retention Time (s)	Energy Consumption (J)	Ref.
Ag/MAPbBr_3_/Pt	100	2000	not given	^38^
Ag/(PEA)_2_Cs_3_Pb_4_I_13_/Pt	230	2000	5.12 × 10^− 10^	^39^
Au/MAPbI_3_/ITO	2300	not given	2.4 × 10^− 10^	^40^
Au/CsPbBr3/ITO	400	500	2.6 × 10^− 6^	^41^
**Al/P3HT/TCP/FTO**	**1000**	**> 1000**	**6 × 10** ^**− 12**^	**This article**

Figure 2a illustrates the measurement of the Al/P3HT/TCP/FTO synaptic device under voltage spikes employing five electrical pulses with an intensity of 1 V, a pulse width and an interval of 35 ms. It is notable that following each stimulation event, the excitatory postsynaptic current (EPSC) level remains elevated in comparison to the presynaptic current. As illustrated in the subsequent formula, the operational energy consumption (E) can be calculated as the product of the pulse voltage (v), pulse current (i), and pulse width (t). In this instance, the resulting value is 6.3 pJ.

1 $$\:E={\int\:}_{0}^{t}i\left(\xi\:\right)v\left(\xi\:\right)d\xi\:$$

This phenomenon demonstrates a degree of persistence and gradually diminishes over time in the absence of consistent pulse voltage stimulation. The decay process of EPSC following stimulus removal is characterised by two distinct stages. Initially, there is a rapid decline, followed by a slower, more gradual decrease. This pattern aligns with the observed forgetting process. As depicted in Fig. 2b, the long-term potentiation (LTP) and long-term depression (LTD) are simulated using two distinct pulse polarities. For the LTP scenario, 50 rectangular pulses with a pulse interval and width of 7 ms and an amplitude of 0.1 V / 1 V were applied, resulting in a highly prominent LTP effect. The trend of synaptic weight increase exhibits a strong linearity, indicating that conductance modulation can be linearly achieved by adjusting the number of pulses.**Figure S2d** demonstrates the LTD phenomenon, where the absolute value of the input current decreases. In this case, an input voltage of -0.6 V / 0.1 V was employed, with rectangular pulses featuring a width and interval of 20 ms. The reduction in synaptic weight follows a logarithmic trend. Furthermore, Fig. 2c illustrates the EPSC curves generated by 2.5 V / 0.1 V voltage pulses. Upon receipt of a positive electrical stimulus, the I-t curve exhibits an immediate rise, followed by a gradual decline to a stable state (depicted in blue). The removal of the stimulus causes the curve to revert rapidly to its original state. This phenomenon can be attributed to the fact that the parasitic capacitance present within the synaptic device cannot be disregarded when the pulse voltage is significant^42^.

Paired-pulse facilitation (PPF) denotes the phenomenon whereby the response to the subsequent stimulus is augmented when neurons transmit nerve impulses^43^. Figure 2**d** provides a further illustration of the extraction of PPF. As the time interval between stimuli increases, the percentage gain from two consecutive stimuli progressively decreases. This behaviour is attributed to the utilisation of smaller pulses, which induce alterations in the accumulation of conductive filaments (CFs), subsequently influencing the effective cross-sectional area of these filaments. The PPF curve can be approximated by the following formula

2 $$PPF = {K_1}{e^{ - \frac{{\Delta t}}{{{\tau _1}}}}} + {K_2}{e^{ - \frac{{\Delta t}}{{{\tau _2}}}}} + {K_0}$$

where K_1_ and K_2_ represent the initial facilitation amounts for the fast and slow phases, respectively. Δt is the time interval between two pulses, and τ_1_ and τ_2_ are the relaxation times for the fast and slow phases, which are measured at 3.28 ms and 30.2 ms, satisfying the plasticity requirements of biological synapses as noted in reference^44^. In the context of LTP, the application of 200 rectangular pulses, with a pulse interval and width of 7 ms and an amplitude of 0.1 V / 1 V, resulted in a markedly pronounced LTP effect. The trend of synaptic weight increase exhibits a strong linearity, indicating that conductance modulation can be achieved by adjusting the number of pulses in a linear manner. Figure 2**e** illustrates the impact of four distinct pulse amplitudes (i.e., pulse voltages) of 0.4 V, 0.6 V, 0.8 V and 1 V, with 23 pulses applied for each amplitude, utilizing data processed with I_n_-I_1_ treatment, with a reading voltage set to 0.1 V. The results demonstrate that the variation in conductivity is significantly influenced by the input voltage amplitude. As the amplitude is increased gradually from 0.4 to 1.0 V, the phenomenon of conductivity enhancement becomes increasingly evident. As the number of pulses increases, it gradually tends towards a saturated state, with the saturation current value varying in accordance with the applied voltage. The amplitude exerts an influence on the electric field strength, which in turn affects the depolarisation level of the p-i-n junction, and thus the modulation effect. This figure illustrates the SVDP of the device. Figure 2f illustrates the modulation effects of three distinct pulse frequencies (70 Hz, 28 Hz, and 15 Hz), with a pulse width-to-gap ratio of 1:1, a pulse voltage amplitude of 1 V, and a reading voltage set to 0.1 V, based on data processed with I_n_-I_1_ treatment. **Figure S3b** shows the I-T curves for the three frequency pulses. The conductance of the device exhibits a frequency-dependent response. This frequency-dependent synaptic behaviour is referred to as synaptic SRDP.

**Fig. 3:**
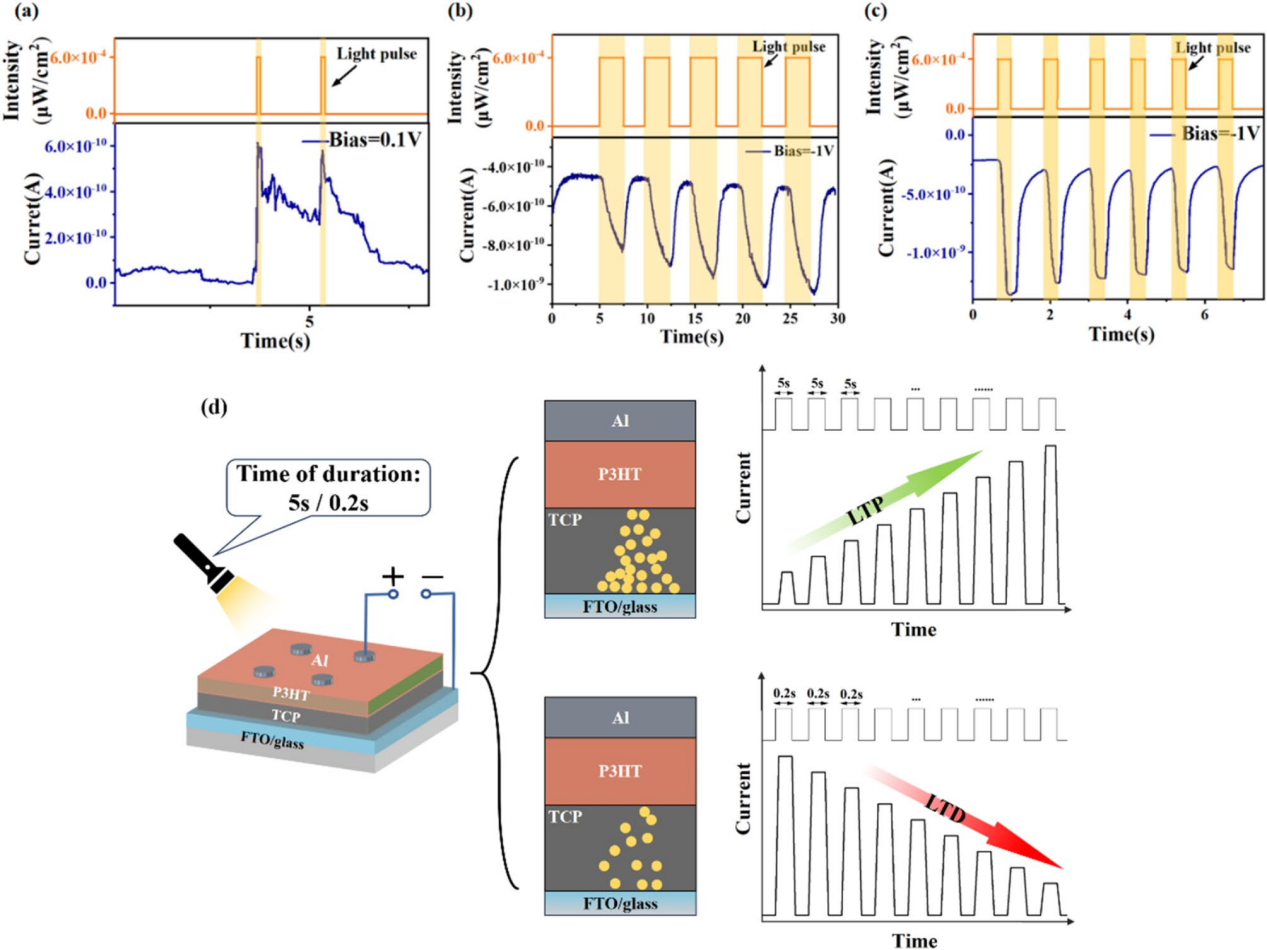
(**a**) Photocurrent response of the Al/P3HT/TCP/FTO device under illumination with a width of 100 ms; (**b**-**c**) Synaptic weight modulation of the Al/P3HT/TCP/FTO device under optical pulses; (**b**) Enhancement with − 1 V bias voltage; (**c**) Suppression with − 1 V bias voltage (where the light source used is 630 nm, 600 µW/cm^2^); (**d**) Mechanisms underlying the generation of LTP and LTD under various duration of light pulses.

The RS effect of the TCP synaptic device exhibits both excellent electrical synaptic features and optical synaptic characteristics, thereby demonstrating its potential for use in visual recognition applications. In this section, the Al/P3HT/TCP/FTO device prepared is employed for the purpose of testing the photoelectric effect. Figure 3 illustrates the EPSC in response to optical pulse stimulation and various sustained electrical excitations (0.1 V and − 1 V). Figure 3**a** illustrates the response of the device to two optical pulses (width ~ 100 ms, wavelength ~ 630 nm, intensity ~ 600 µW/cm^2^) with a continuous application of a reading voltage of 0.1 V, which results in a significant sudden increase in current. This demonstrates that the synaptic device can effectively respond to light signals. Following the cessation of the optical stimulation, the EPSC current of the device continues to decay until the next pulse is applied. The operational energy consumption can be calculated as the product of pulse voltage, pulse current, and pulse width, which in this context amounts to 6 pJ. Furthermore, our experiments indicate that varying pulse frequency and bias voltage can lead to both the enhancement and suppression of simulated synaptic weights. As illustrated in Fig. 3b, the application of a bias voltage of -1 V in conjunction with larger square light pulses (5 s width, light period approximately 10 s) results in a gradual and stepwise increase in the generated photocurrent. This specific configuration effectively emulates the complex learning processes observed in the human brain. With each additional learning cycle, the excitability of the system is enhanced, resulting in the formation of more robust memories. Conversely, as illustrated in Fig. 3c, when the bias voltage is maintained at -1 V, the application of narrower square light pulses (0.2 s width, light period approximately 1.2 s) results in a gradual attenuation of the generated photocurrent, thereby demonstrating the inhibitory effect on synaptic weight. The underlying mechanisms of the generation of different currents are illustrated in Fig. 3d. At a bias of 0.1 V, the photocurrent undergoes an instantaneous surge because the applied voltage is lower than the voltage that switches the memristor to a low-resistance state, thereby favoring thermally generated free carriers over those injected at low voltage. However, when light stimulation occurs at biases of -1 V, the current increase is characterised by quadratic upward and downward trends. This behaviour arises from the formation of a relatively stable conductive filament channel, where the proportion of photoinduced carriers introduced by light^45^ is comparatively modest. Furthermore, it can be observed that the proportion of injected photoinduced carriers, and consequently the enhancement or suppression of EPSC, can be effectively modulated by varying the pulse width of light stimulation.

**Fig. 4:**
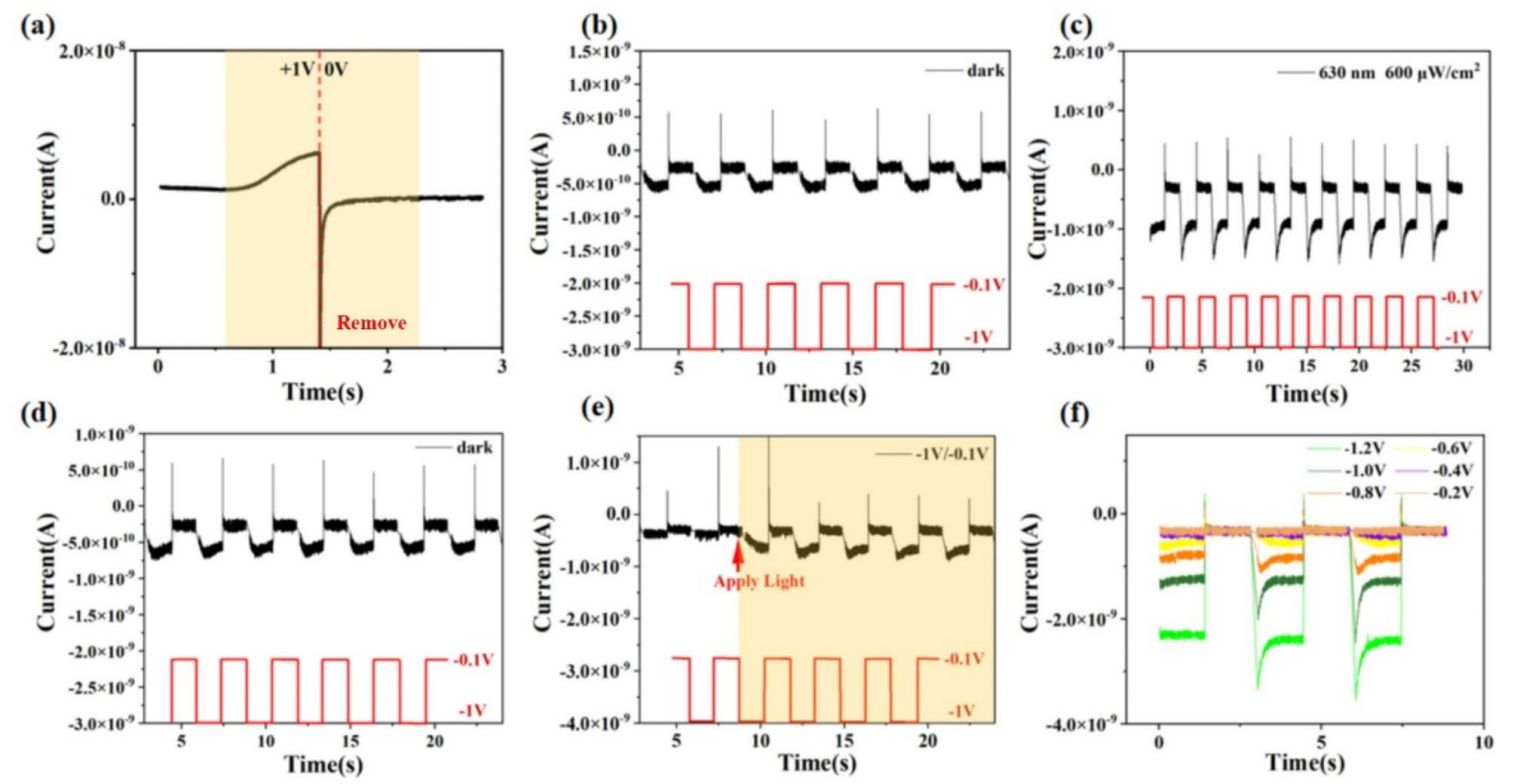
(**a**) Photolithography-assisted electrical erasure process, implemented by writing with light and erasing with 0 V; (**b**) Under voltage inputs of -1 V / -0.1 V (as indicated by the blue rectangle), first measure the dark-state current-time curve, (**c**) EPSC under illumination with 630 nm, 600 µW/cm^2^, (**d**) EPSC by removing the illumination for a dark-state measurement. (**e**) Apply illumination during scanning in the dark state. (**f**) Apply six different sizes of voltage stimuli − 0.2 V / -0.1 V, -0.4 V / -0.1 V, -0.6 V / -0.1 V, -0.8 V / -0.1 V, -1.0 V / -0.1 V, -1.2 V / -0.1 V under illumination at 630 nm and 600 µW/cm^2^.

Following the demonstration of light pulse modulation of synaptic plasticity under various voltages, further research has been conducted on the optoelectronic cooperative control. As illustrated in Fig. 4a, the inscription of information is achieved through the application of light at a bias voltage of 1 V, resulting in a gradual increase in current. Subsequently, the EPSC can be removed, specifically by adjusting the input voltage to 0 V for erasure, thereby achieving the synergistic function of light-induced writing and electrical erasure. Subsequently, a study on the modulation of EPSC by varying light pulses under fixed electrical stimuli was presented in Fig. 4b-d. Initially, a square wave electrical stimulus with a voltage of -1 V, pulse intervals and widths of approximately 1.5 s, was applied in a dark environment. The EPSC initially increases and subsequently reaches a stable equilibrium (Fig. 4**b**). Subsequently, a significant alteration in the current waveform was observed when the device was continuously exposed to light with a wavelength of 630 nm and an intensity of 600 µW/cm^2^ (Fig. 4**c**). It is noteworthy that following the removal of light exposure, the resistive synaptic device is still able to return to its initial state (Fig. 4**d**). To further verify the reproducibility of the aforementioned phenomena, as illustrated in Fig. 4e, three sets of electrical stimuli with a potential of -1 V and − 0.1 V, respectively, were applied in the absence of light, followed by continuous exposure to light. It can be observed that the application of light has no discernible impact on the current at -0.1 V. However, the current during the − 1 V voltage period exhibits a notable increase following light exposure, reaching from − 5 × 10^− 10^ A to -1.5 × 10^− 9^ A, which is consistent with the phenomenon depicted in Fig. 4c. Moreover, the regulations governing optoelectronic cooperative control in response to disparate voltage pulse stimuli were examined in Fig. 4f. Six voltage pulses with varying amplitudes of -0.2 V, -0.4 V, -0.6 V, -0.8 V, and − 1 V are applied while the light source remains at a wavelength of 630 nm and intensity of 600 µW/cm^2^. It can be observed that when the pulse voltage is less than 0.4 V, the application of light does not elicit a notable response. As the voltage exceeds 0.6 V, the photoelectric coupling effect becomes increasingly evident, exhibiting a progressive intensification with rising pulse voltage. The current declines exponentially as capacitance charging diminishes over time, analogous to the brain gradually becoming more tranquil from initial excitement. The potential of photoelectric resistive switching devices for use in optical-coupling synapses is promising, with the possibility of enhancing artificial neural networks for faster data storage and retrieval.

**Fig. 5:**
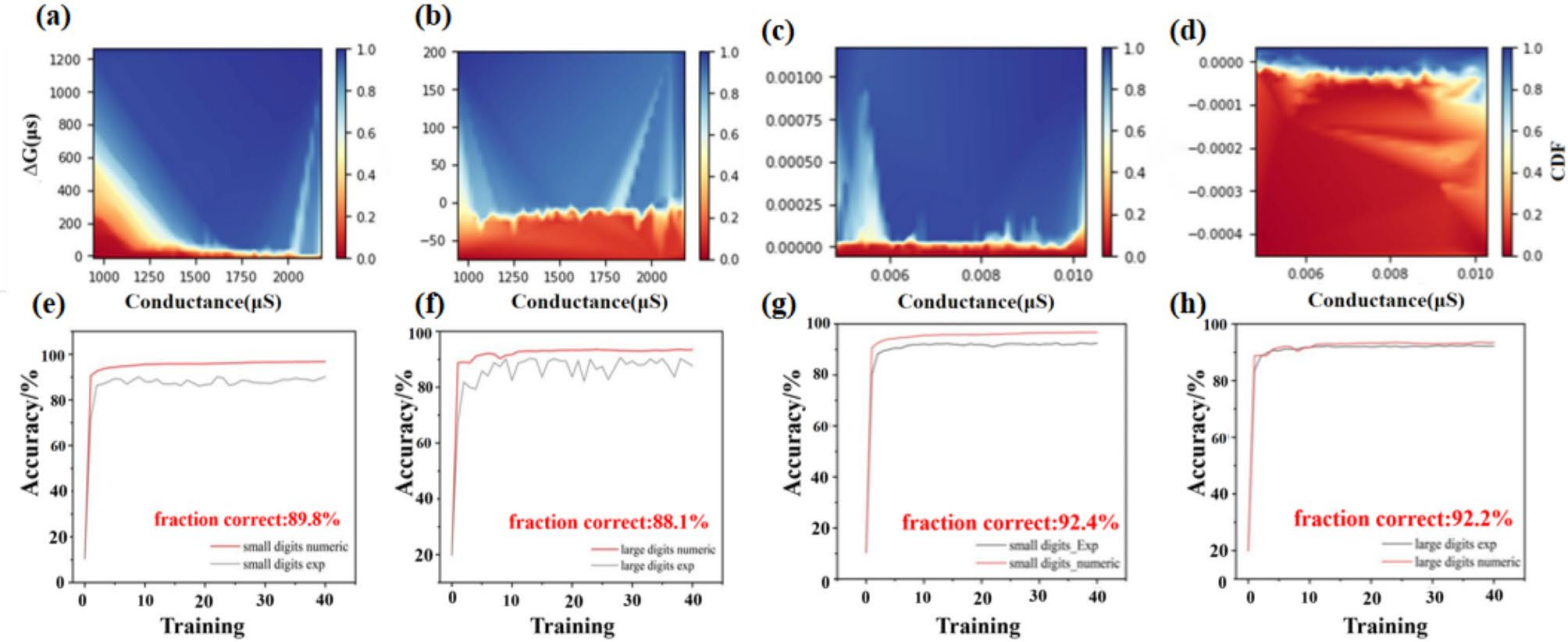
(**a**-**b**) Utilizes electrical synaptic characteristic data image recognition simulation during the enhancement and suppression periods, ∆G’s CDF; (**c**-**d**) Uses optical synaptic characteristic data image recognition simulation during the enhancement and suppression periods, ∆G’s CDF; (**e**-**h**) Recognition accuracy for different tasks, small images (64 × 36 × 10), large images (784 × 300 × 10).

In accordance with the aforementioned photoelectric response characteristics, arithmetic operations, and Pavlovian conditioned reflex experiments, the successive implementation of simulation and experimentation is demonstrated in **Figures S2-4**. Furthermore, a comparison is presented between the image processing capabilities of pure electric and optoelectronic cooperative mechanisms. Figure 5**a-b** illustrates the cumulative distribution functions (CDF) of ∆G for LTP/LTD, which determine the magnitude of the write noise based on their average update noise values. Figure 5**e-f** illustrate the synaptic weight mapping images of a neural network for small (8 × 8 handwritten digit dataset) and large (28 × 28 handwritten digit dataset) images, respectively, after 10 learning training stages. The image data is converted to grayscale values (0 ~ 255) and subsequently transmitted to the input layer. Following ten learning stages, the respective recognition accuracies were found to be 89.8% and 88.1%. With regard to the optoelectronic cooperative control mechanism, Fig. 5c-d illustrate the CDF of the learning rate for LTP and LTD, respectively, and determine the size of the write noise based on them. As illustrated in Fig. 5g-h, the results demonstrate that synaptic weight mapping images in the neural network for the same images attain stable recognition accuracies of 92.4% and 92.2%, respectively.

## Conclusion

In conclusion, the incorporation of a P3HT modifying layer onto triple cation halide perovskite thin films has proven to be an effective method for enhancing the RS characteristics of synaptic devices. The resulting synaptic device exhibits commendable stability, with 103 endurance cycles and > 10^3^ s retention time, along with low energy consumption (~ 6.3 × 10^− 12^ J for electrical stimulus and ~ 6 × 10^− 12^ J for optical stimulus). Furthermore, the synaptic characteristics of the perovskite/P3HT heterojunction synaptic device were examined under optoelectric coordinated modulation, which encompassed LTP, LTD, SRDP, and SVDP. The linear characteristics of synaptic plasticity enable the synaptic device to perform arithmetic operations, Pavlovian conditioned reflexes, and vision recognition. It is noteworthy that the recognition accuracies of 89.8% / 88.1% (electric synapse) are significantly enhanced to 92.4% / 92.2% following the introduction of optoelectronic cooperative stimulation on the 8 × 8 and 28 × 28 MNIST handwritten digit datasets. The findings of this research have significant implications for the guidance of optoelectronic co-regulation of perovskite synaptic devices within the domain of synaptic electronics.

## Experimental section

### Device fabrication

**Fig. 6:**
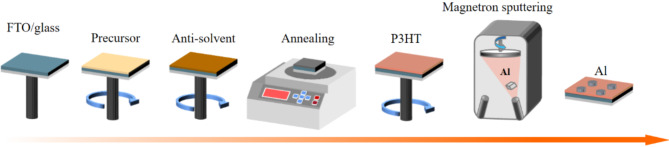
Fabrication process of Al/P3HT/TCP/FTO synaptic device.

The fabrication process of the Al/P3HT/TCP/FTO synaptic device is depicted in Fig. 6. The substrate is a 15 mm×15 mm×1.6 mm single-sided FTO-coated glass, which serves as the bottom electrode. The FTO glass was positioned on a PTFE glass rack within a beaker and processed in successive steps using a glass cleaning agent solution, deionized water, and anhydrous ethanol. Subsequently, the FTO surface was subjected to a 10-minute UV-ozone plasma cleaning process, which served to eliminate organic substances, enhance substrate hydrophilicity and facilitate solution spreading during the subsequent spin-coating process. The synthesis of the CsFAMAPbI_x_Br_3−x_ precursor involved the use of 15.2 mg CsI, 173.5 mg FAI, 19.9 mg MABr, 65.4 mg PbBr_2_, and 490.1 mg PbI_2_. Subsequently, a volume of 35 µL of the precursor solution was pipetted onto the FTO substrate using a two-step spin-coating method. The initial stage of the process entails a rotation speed of 2000 rpm for a period of 10 s, followed by a second stage with a rotation speed of 4000 rpm for 30 s. In the final 11 s of the complete spin-coating process, 220 µL of chlorobenzene (CB) was added uniformly in dropwise fashion using a pipette as an antisolvent. Subsequently, P3HT was spin-coated onto the perovskite film using the aforementioned method. Subsequently, a pure Al electrode was deposited onto the perovskite/P3HT heterojunction by magnetron sputtering using an Al target under a gas flow of Ar (99.995% purity with a total flux of 60 sccm). The thickness of the Al electrode was measured by atomic force microscopy (AFM) and found to be 50 nm.

### Device characterization

A XRD test was conducted using a Cu Kα radiation X-ray diffractometer (X’pert PRO MPD) with a wavelength of 1.54056 Å. The XRD data were collected in the 5° to 50° range, with a step scan size of 0.02° and a scan speed of 0.2°/s. Cross-section SEM images were obtained using a high-resolution instrument (APREOS, Thermo Fisher Scientific). The XPS image was characterised using an Alpha300R confocal Raman spectrometer. The surface of the TCP/P3HT film was examined using atomic force microscopy (MFP-3D-BIO). The electrical performance was evaluated using a Keysight B2902A semiconductor parameter analyser.

## Electronic supplementary material

Below is the link to the electronic supplementary material.

Supplementary Material 1

## Data Availability

The data that support the findings of this study are available from the corresponding author upon reasonable request.
