# Supplementary material for: Optoelectric coordinated modulation of resistive switching behavior in perovskite based synaptic device
Source: Sci Rep. 2025 Feb 7;15:4626. doi: 10.1038/s41598-025-88716-8 (PMC11805995; doi:10.1038/s41598-025-88716-8)
Supplement: Supplementary file 1 — Supplementary Material 1 [file 41598_2025_88716_MOESM1_ESM.docx]

**Modulation and Applications of Resistive Switching Behavior in Perovskite Resistive Memory Devices under Light Stimulation**

Yucheng Wang^a1^, Ruixi Huang^a2^, Wenyi Zhang^2^, Dingyun Guo^2^, Jiawei Zheng^2^, Hexin Wang^2^, Fobao Huang^2^, Zhuoya Wang^2^, He Guan*^2^

^1^Research&Development Institute of Northwestern Polytechnical University in Shenzhen

^2^School of Microelectronics, Northwestern Polytechnical University, Xi’an, 710072,

China.

*Corresponding author. E-mail: he.guan@nwpu.edu.cn

^a^These authors contributed equally to this work.


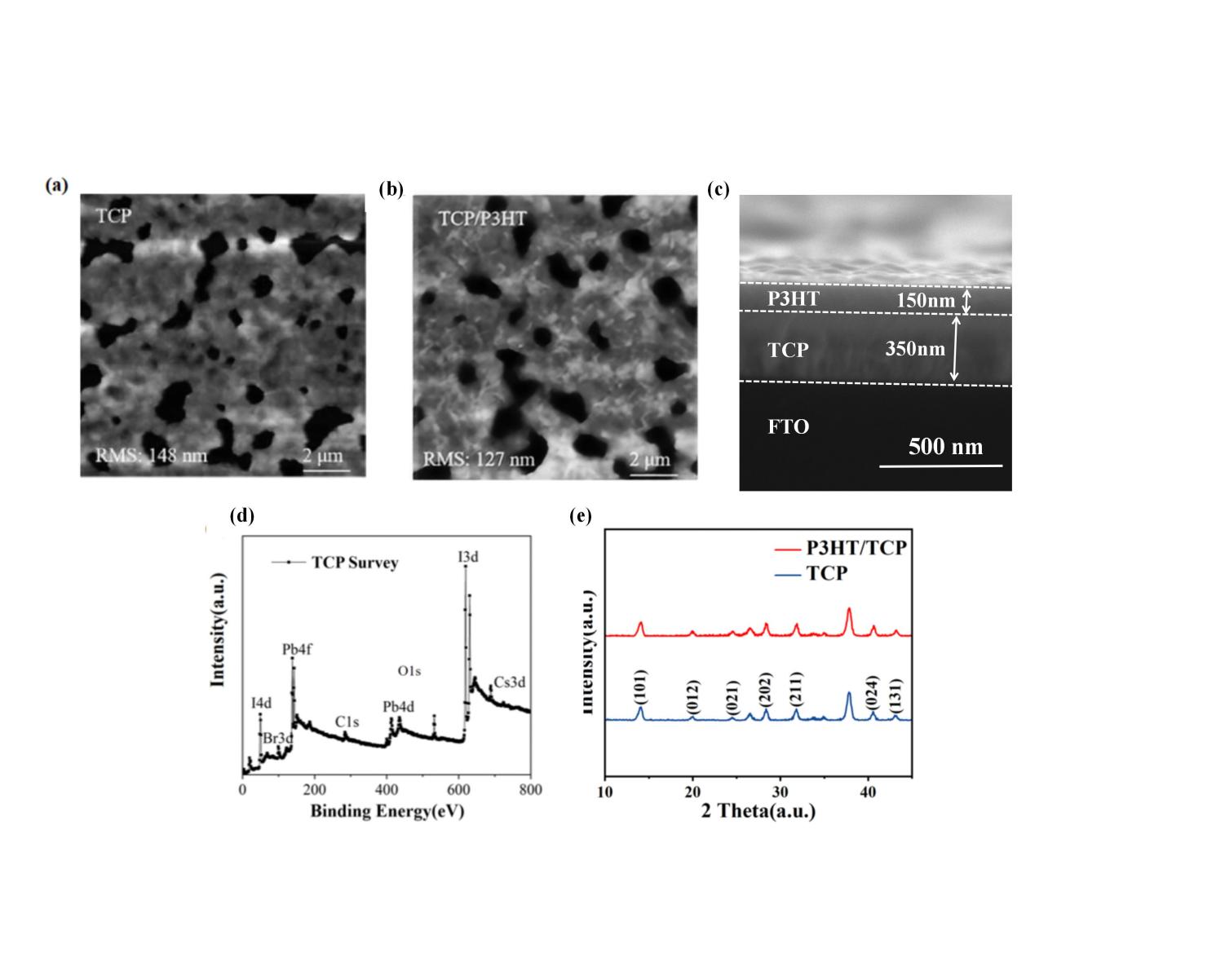


**Figure S1** (a-b) Atomic force microscope (AFM) images of the TCP and TCP/P3HT layer; (c) Cross-sectional SEM images of the TCP/P3HT layers; (d) The X-ray photoelectron spectroscopy (XPS) spectra of the TCP surface layer; (e)XRD spectra of the TCP and TCP/P3HT films.

Atomic force microscope (AFM) images of the TCP and TCP/P3HT layer are shown in **Figure S1a-b**. An analysis of the SEM image in **Figure S1c** indicates that the TCP and P3HT films have approximate thicknesses of 350 nm and 150 nm, respectively. The XPS spectra of the TCP/P3HT film exhibited the following peaks: I4d (~49 eV), Br3d (~68 eV), Pb4f (~138 eV), C1s (~284 eV), Pb4d (~413 eV), I3d (~619 eV), and Cs3d (~724 eV), which is shown in **Figure S1d**. **Figure S1e** illustrates the X-ray diffraction (XRD) spectra of TCP and TCP/P3HT hybrid layers.


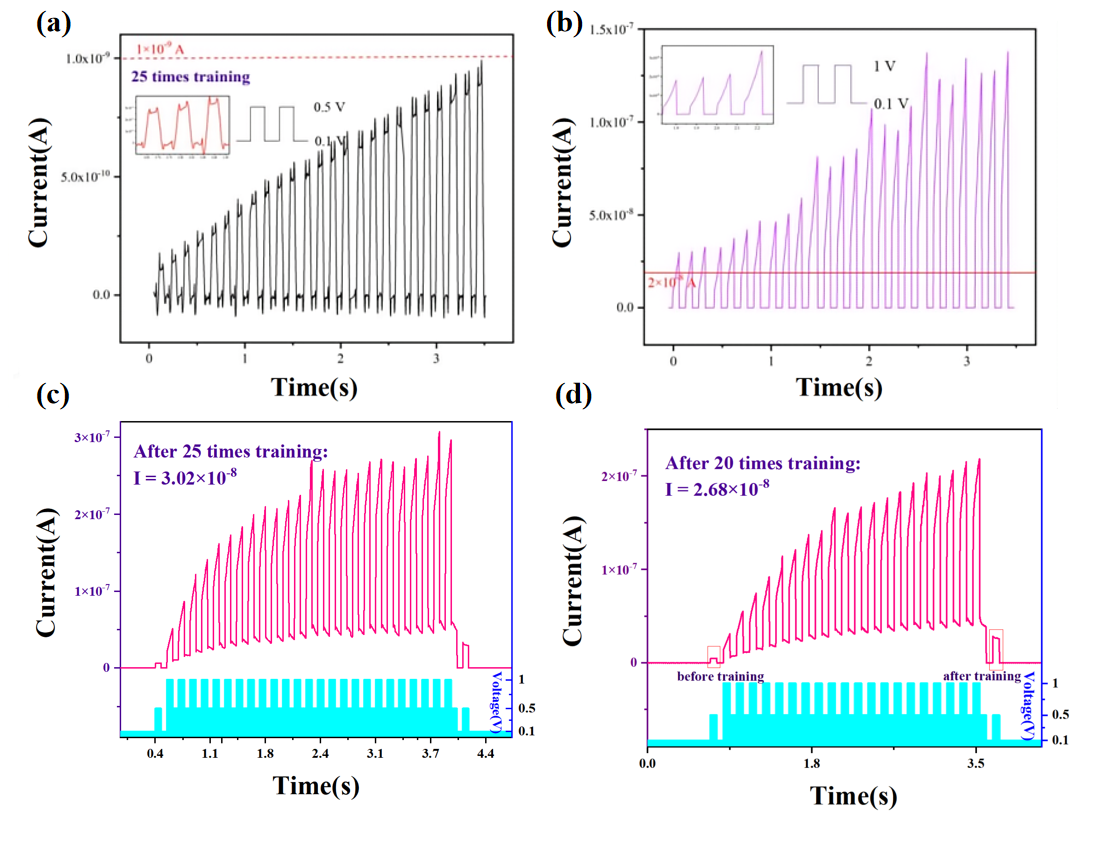


**Figure S2** Simulating Pavlov's dog experiment: (a) Stimulation with a 'bell' signal (25 continuous pulses of 0.5V/0.1V voltage, width and interval of 70 ms); (b) Stimulation with a 'meat' signal (25 continuous pulses of 1V/0.1V voltage, width and interval of 70 ms); (c) 20 combined stimulations of 'meat' and 'bell' signals, with a pause in voltage after one pulse interval, followed only by the 'bell' signal; (d) 25 combined stimulations of 'meat' and 'bell' signals, with a pause in voltage after one pulse interval, followed only by the 'bell' signal.

Pavlov's dog experiment is one of the most famous examples of classical conditioning. In **Figure S2**, this experiment is simulated using FTO/TCP/P3HT/Al memristors. Assuming a threshold current of 2×10^-8^ A, a 0.5V pulse voltage with a width and interval of 70 ms simulates the 'bell' signal (see **Figure S2a**). It takes 25 'bell' signal currents to reach a basic level of 1×10^-9^ A, far below the excitatory threshold (not excited). Using 1V pulse voltage with the same width and interval simulates the 'meat' signal (see **Figure S2b**), where the first pulse reaches the threshold current and triggers a response (excited). Training with a combination of 'meat' and 'bell' signals for associative learning (see **Figure S2c-d**) demonstrates that by alternately applying 'meat' and 'bell' signals through repetitive cycles, reaching the threshold current (2×10^-8^ A) can be achieved solely through the 'bell' signal, inducing excitement. The experimental results indicate that the degree of excitement is related to the number of training sessions. After 20 (**Figure S2c**) and 25 (**Figure S2d**) training sessions, immediately after removing the voltage for one pulse interval, the responses to the individual 'bell' stimuli reached 2.68×10^-8^ A and 3.02×10^-8^ A, respectively. This illustrates that after removing the 'meat' signal, the 'bell' signal can evoke an excited state, indicating established associative learning. Furthermore, as the number of training sessions increases, the level of excitement grows stronger. In **Figure S3**, seven sets of alternating pulse stimuli identical to those in **Figure 5** were applied, and the dashed lines represent the current connection forming associative memories for individual 'bell' stimuli after each training session. The experimental results show that with an increase in the number of training sessions, the level of excitement gradually increases and tends to saturate after five sessions.


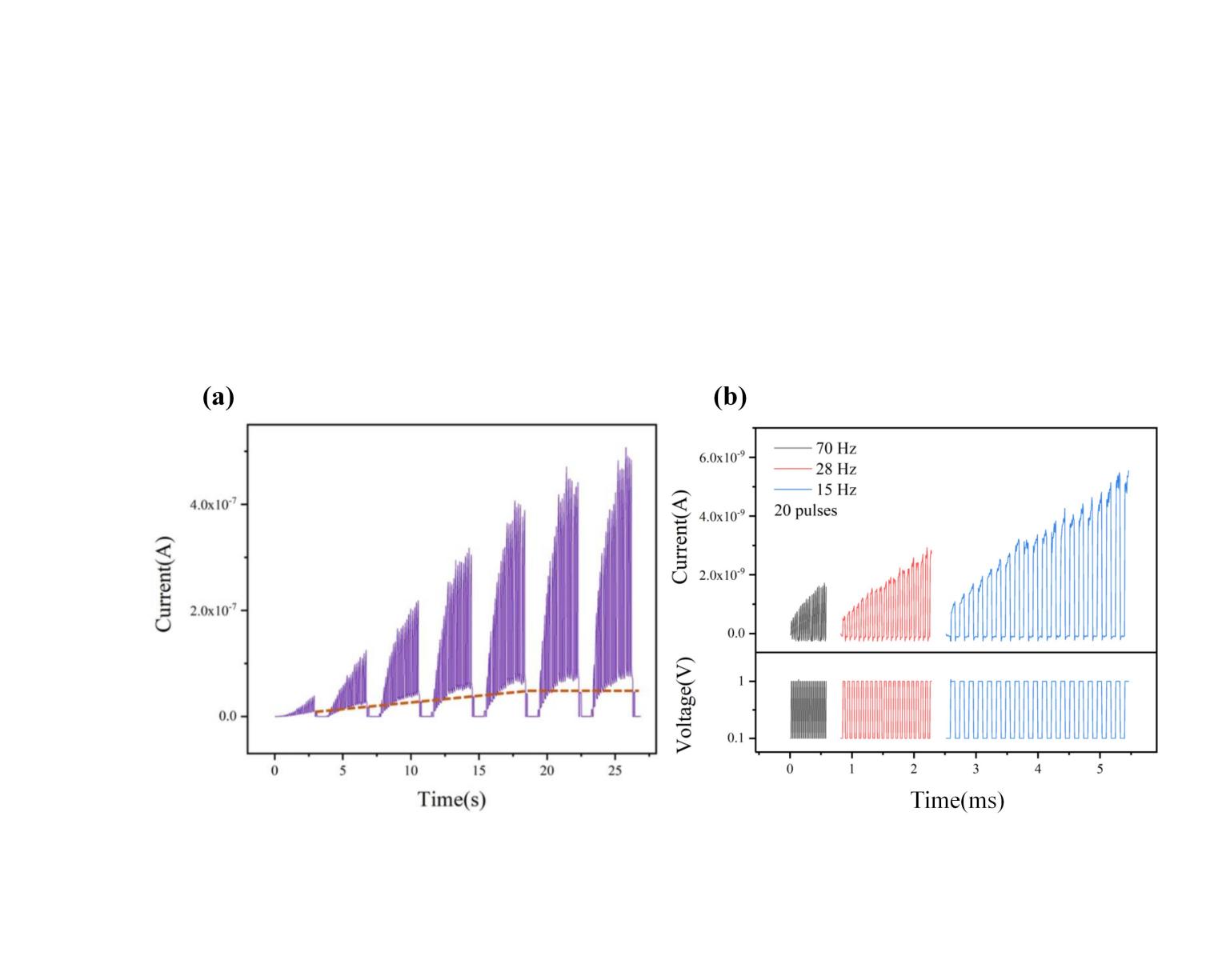


**Figure S3** (a) Experimental results of multiple sets of associative memory training, each comprising 20 electrical pulses, are consistent with the pulses used in **Figure S2**; (b) Modulation effect at different pulse frequencies with 20 pulses, each at 1 V amplitude, and a read voltage of 0.1 V (I-T test results)


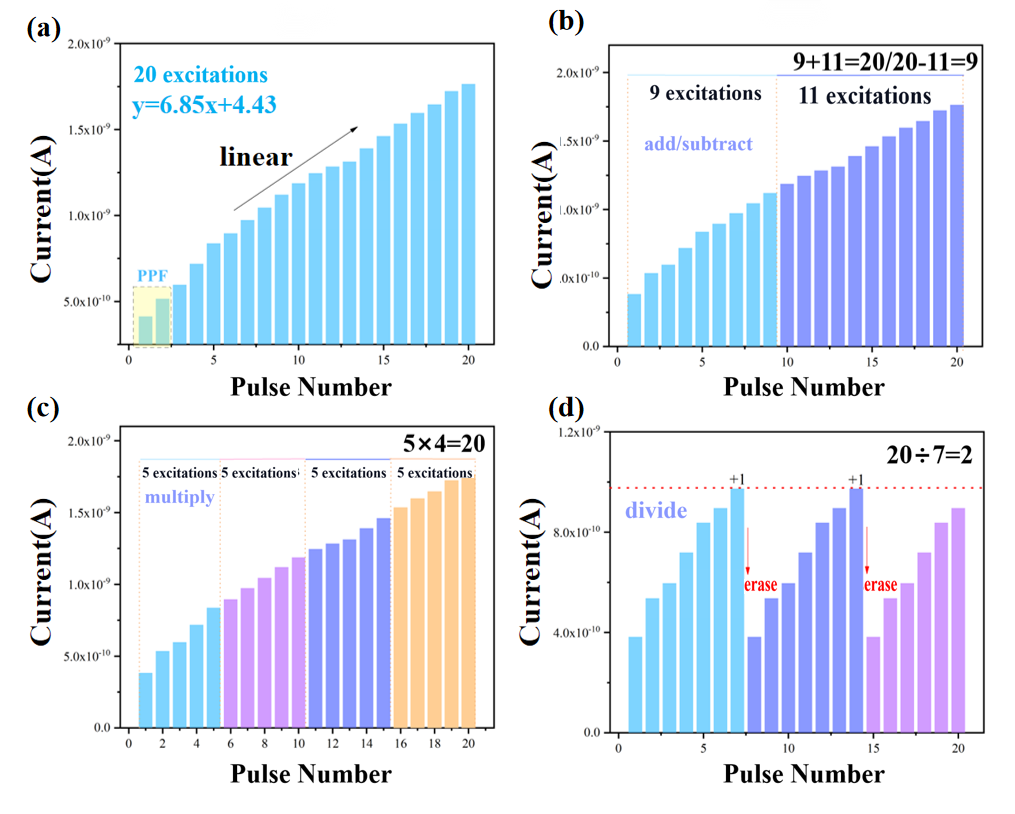


**Figure S4** (a) The first 16 pulse curves of the device achieving LTP under 0.1 V/1 V electrical pulses, with numbers assigned from 1 to 20 corresponding to the respective LTP; (b-d) Perform exponential addition/subtraction (b), multiplication (c), and division (d) arithmetic calculations, respectively.

The data extracted in **Figure S4** is from the electrical LTP-related data of the Al/P3HT/TCP/FTO device of the previous section (applying pulse voltage of 1 V/0.1 V), demonstrating a good linear rising trend and the potential for logic operations. As shown in **Figure S4a**, the current-count data for the first 20 pulses are selected and fitted into a binary linear function: y = 6.85x + 4.43. The yellow box in the figure indicates its evident PPF and PTP characteristics. The current values are selected at the maximum EPSC, and due to its good linearity, the current induced by the nth rectangular voltage can be allocated to a specific number n within the range of 1 to 20. A one-to-one relationship between n and In is established for logic arithmetic simulation. **Figure S4b** displays simulated addition/subtraction operations, where the continuous voltage-induced current can be matched with a specific I_n+m_ to calculate the sum of numbers. Subtracting n from m can also be calculated by examining the number of light pulses required for the current to increase from In to Im. In the example in the figure, 9 consecutive light pulses, followed by 11 consecutive light pulses, result in a current of approximately 1.77 × 10^-9^ A, corresponding to the current I20 in the twentieth pulse. This allows the calculation of 9 + 11 = 20 or 20 - 11 = 9, achieving addition or subtraction calculations. **Figure S4c** simulates multiplication operations, where in the example, four sets of consecutive 5 pulses result in the current corresponding to I20 (1.77 × 10^-9^ A), simulating the multiplication operation 5 × 4 = 20. **Figure S4d** simulates division operations, setting In as the threshold, counting up by 1 each time the current reaches In, using reverse voltage erasure to reset the current to I_0_, repeating this process until the pulse count reaches m. In the example, when the current reaches I_7_, approximately 9.74 × 10^-10^A, the count increases by 1, and the process continues until the pulse count reaches 20, simulating 20 ÷ 7 = 2 (no rounding in computer programming language division operations).
